# Characteristics of the tree shrew gut virome

**DOI:** 10.1371/journal.pone.0212774

**Published:** 2019-02-26

**Authors:** Linxia Chen, Wenpeng Gu, Chenxiu Liu, Wenguang Wang, Na Li, Yang Chen, Caixia Lu, Xiaomei Sun, Yuanyuan Han, Dexuan Kuang, Pinfen Tong, Jiejie Dai

**Affiliations:** 1 Center of Tree Shrew Germplasm Resources, Institute of Medical Biology, Chinese Academy of Medical Sciences and Peking Union Medical College, Yunnan Key Laboratory of Vaccine Research and Development on Severe Infectious Diseases, Yunnan Innovation Team of Standardization and Application Research in Tree Shrew, Kunming, China; 2 Department of Pathogenic Biology, School of Basic Medical Science, Gannan Medical University, Ganzhou, China; 3 Department of Acute Infectious Diseases Control and Prevention, Yunnan Provincial Centre for Disease Control and Prevention, Kunming, China; 4 MOE Key Laboratory of Bioinformatics and Bioinformatics Division, Center for Synthetic and System Biology, TNLIST/Department of Automation, Tsinghua University, Beijing, China; Arizona State University, UNITED STATES

## Abstract

The tree shrew (*Tupaia belangeri*) has been proposed as an alternative laboratory animal to primates in biomedical research in recent years. However, characteristics of the tree shrew gut virome remain unclear. In this study, the metagenomic analysis method was used to identify the features of gut virome from fecal samples of this animal. Results showed that 5.80% of sequence reads in the libraries exhibited significant similarity to sequences deposited in the viral reference database (NCBI non-redundant nucleotide databases, viral protein databases and ACLAME database), and these reads were further classified into three major orders: *Caudovirales* (58.0%), *Picornavirales* (16.0%), and *Herpesvirales* (6.0%). *Siphoviridae* (46.0%), *Myoviridae* (45.0%), and *Podoviridae* (8.0%) comprised most *Caudovirales*. *Picornaviridae* (99.9%) and *Herpesviridae* (99.0%) were the primary families of *Picornavirales* and *Herpesvirales*, respectively. According to the host types and nucleic acid classifications, all of the related viruses in this study were divided into bacterial phage (61.83%), animal-specific virus (34.50%), plant-specific virus (0.09%), insect-specific virus (0.08%) and other viruses (3.50%). The dsDNA virus accounted for 51.13% of the total, followed by ssRNA (33.51%) and ssDNA virus (15.36%). This study provides an initial understanding of the community structure of the gut virome of tree shrew and a baseline for future tree shrew virus investigation.

## Introduction

The tree shrew (*Tupaia belangeri*) belongs to the family Tupaiidae, order Scandentia, which has a wide distribution in South Asia, Southeast Asia and Southwest China [1]. The tree shrew is a small mammal similar in appearance to squirrels and feeds on fruits, insects and small vertebrates [2]. *Tupaia belangeri* is the only representative in China and consists of six subspecies: *T*. *belangeri gaoligongensis*, *T*. *belangeri modesta*, *T*. *belangeri yaoshanensis*, *T*. *belangeri tonquinia*, *T*. *belangeri yunalis* and *T*. *belangeri chinensis* [3]. Previous studies [4–5] showed that the tree shrew has a closer relationship with humans than did rodents in terms of physiological function, biochemical metabolism and genomic signatures. Due to its unique characteristics, such as small body size, low cost of maintenance, life span and short reproductive cycle, the tree shrew has been increasingly used in laboratory analyses in recent years. Several studies have used this animal for the construction of human disease models, such as models for hepatitis virus, influenza virus, cytomegalovirus, herpes simplex virus, and dengue virus [6–8].

Although some viruses were isolated or detected from tree shrew in previous reports, the gut viral diversity for this animal is still unknown. In addition, traditional methods, such as cell culture or PCR, failed to fully estimate the distribution of microorganisms and taxonomic diversity. However, the recent availability of next-generation sequencing methods has provided a thorough investigation of the complex and diverse gut virome, and a large number of gut metagenomics studies have been conducted [9–11]. Previous gut virome analysis mainly referred to human or other animals [12–13], not including the tree shrew. Because of the importance of zoological research, it is necessary to determine the gut virome of this animal. Therefore, in this study, a viral metagenomic method based on next-generation sequencing was used to reveal the characteristics of the gut virome for tree shrew collected from the suburbs of Kunming, China.

## Materials and methods

### Sample source and preparation

Fifty fecal samples from tree shrews were collected at the Center of Tree Shrew Germplasm Resources, Institute of Medical Biology, Chinese Academy of Medical Science and Peking Union Medical College in Kunming, China (103°40′ E, 26°22′ N). All the animals were housed for use in further research without any sacrifice. Fresh feces were collected and immediately stored at -70°C. The animals were healthy without visible features of tumors or disease; 30 were male, 20 were female, and the average weight was 130.25±18.76 g. Each sample was resuspended in sterile phosphate-buffered saline (PBS), centrifuged at 8,000 rpm (15 min, 4°C), and then filtered through a 0.45 μm and 0.22 μm syringe filter (Millipore, Bedford, MA). All samples were pooled and ultra-centrifuged at 40,000 rpm for 4 h at 4°C. Subsequently, the supernatant was discarded, and the pellet was resuspended in 500 μl PBS and then treated with a cocktail of DNase (TaKaRa Bio Inc. Japan), benzonase (TaKaRa Bio Inc. Japan) and RNase (TaKaRa Bio Inc. Japan) [14].

### Nucleic acid extraction and sequence-independent amplification

Viral nucleic acids were extracted from nuclease-treated resuspended supernatant by using a QIAamp Viral RNA Mini Kit (QIAGEN, Germany) and OMEGA E.Z.N.A Viral DNA Kit (OMEGA, USA) following the manufacturer’s instructions. A NanoDrop spectrophotometer (Thermo Scientific, USA) was used for the quantification of viral nucleic acids. The extracted viral RNA was reverse transcribed with the PrimeScript II 1st Strand cDNA Synthesis Kit (TaKaRa Bio Inc. Japan) using the primer R-6N (GCCGGAGCTCTGCAGATATCNNNNNN). Then, the second-strand synthesis was run for 1 h at 37°C with Klenow fragment (3’-5’exo-, NEB, Ipswich, MA) into double-strand DNA [14]. Sequence-independent amplification was performed using the primer R (GCCGGAGCTCTGCAGATATC), and the amplification procedures were 94°C for 10 min, followed by 35 cycles of 94°C for 40 s, 55°C for 40 s, 72°C for 90 s, and finally 72°C for 10 min. The primer sequence was cut off with *EcoRV* (TaKaRa Bio Inc. Japan), and the product was purified using a QIAquick PCR Purification Kit (QIAGEN, Germany). The purified product was electrophoresed on a 1% agarose gel.

### Next-generation sequencing and bioinformatics analysis

Sequencing libraries were generated using the NEBNext Ultra DNA Library Prep Kit for Illumina (NEB, USA) following the manufacturer’s recommendations, and index codes were added to the sample. Briefly, the DNA sample was fragmented by sonication to a size of 300 bp, and then the fragmented DNA was end-polished followed by A-tailed ligation with the full-length adaptor for Illumina sequencing with further PCR amplification. The libraries were analyzed by an Agilent 2100 Bio-analyzer and quantified using real-time PCR. Sequencing was performed on an Illumina HiSeq2500 platform, and paired-end reads were generated. Sequence data were deposited in the NCBI database with the SRA accession SRP154022.

The raw reads were quality controlled by removing low-quality sequences, adapters, primers and host sequences. Briefly, low sequencing quality reads were trimmed using Phred quality score 10 as the threshold. Adaptor and primer sequences were trimmed by using the default parameters of QIIME [15]. Host reads and bacterial reads were subtracted by mapping the reads to tree shrew reference genome [16] (accession number deposited at GenBank: ALAR00000000) and bacterial RefSeq genomes release 59 using bowtie2 [17]. The filtered, clean data were aligned and compared to the NCBI non-redundant nucleotide databases (Version: 2014-10-19), reference viral protein databases (Refseq version: 2015-09-08) and ACLAME database (Viruses) using tBLASTx, BLASTn and BLASTx to identify the reads identity [18–20]. BLAST hit with significant E-value was reported by using a threshold E-value of ≤10^−3^, and given the similarity was higher than 75% [21–22]. Several related taxonomies were yielded through an equally high scoring top hit, and these reads were assigned to most recent common ancestor. Reads which did not match genomes used in the clean data and did not match viral genomes included in the database were reported as of unknowns (others). The taxonomies of the aligned reads based on the hit sequence match from all lanes were parsed by Krona [23].

### Phylogenetic analysis

Based on the sequence results mentioned above, specific primers were designed to detect adenovirus in this study. The PCR primers were designed with Clone Manager Professional Suite 8 software (Scientific & Educational Software) (Table 1). The adenovirus *3’UTR* gene was amplified by using primers F1/R1 for the first round and F2/R2 for the second round. The amplified products were sent for bidirectional sequencing, merged by DNAStar software (Lasergene) and compared to the NCBI database using BLASTn. The aligned sequences were trimmed to match the genomic regions of the sequences obtained in this study and to generate phylogenetic trees in MEGA 6.0 [24] using the neighbor joining method with 1000 bootstrap replicates.

**Table 1:** The PCR primers used in this study.

Genes	Primers	Sequences (5'-3')	Amplification length
*UTR*	F1	CGTGCTTTACACGGTTTTTGA	316 bp
	R1	GGTACCTTCAGGACATCTTTGG	
	F2	ACGGTTTTTGAACCCCACAC	
	R2	GTCCTTTCGGACAGGGCTTT	

The perspective phylogenetic analysis of some top virus species was performed by extracting the best match sequences from total valid reads using CLC Genomics Workbench 9.5.2 (QIAGEN, Denmark). The reference genomes of *Cercopithecine herpesvirus 5* (accession: NC_012783), *Theilovirus* (accession: NC_001366), *African bat icavirus A* (accession: NC_026470), and *Cosavirus JMY-2014* (accession: NC_025961) were used. The contigs were assembled and generated from joining overlapped reads from different pairs. All the reads were aligned to each reference genomes by CLC Genomics Workbench 9.5.2 (QIAGEN, Denmark) with the default parameters. We selected parts of the regions of sequences for each contig to build the phylogenetic trees with MEGA 6.0 mentioned above.

### Ethics approval statement

The sample collection and detection protocols were carried out in accordance with relevant guidelines and regulations approved by the Ethics Committee of the Institute of Medical Biology, Chinese Academy of Medical Sciences and Peking Union Medical College. All experimental procedures were approved by the Ethics Review Committee [Institutional Review Board (IRB)] of the Institute of Medical Biology, Chinese Academy of Medical Sciences and Peking Union Medical College.

## Result

### Sequencing data analysis

**Fig 1:**
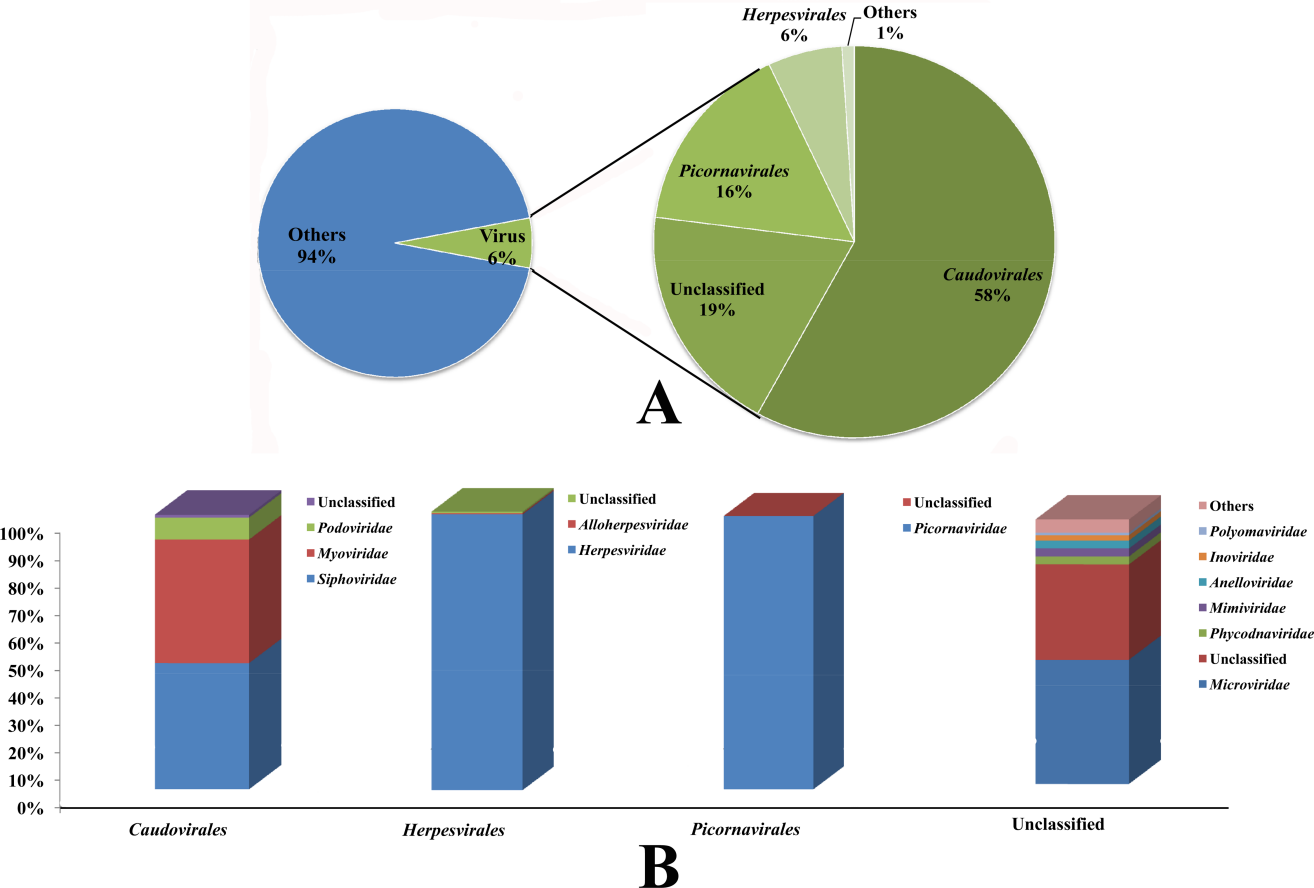
Taxonomic distributions of the virus-related sequences of the tree shrew gut virome. A. The left pie-chart showed the reads alignment results; the right one indicated the taxonomic distributions of orders; B. The proportions of taxonomic distributions of families for each order.

A total of 20,260,886 raw reads were generated, and 20,195,972 reads were validated after trimming and removing adapter and host genomic sequence. The data output for the clean data was 3,033.48 Mbp; 3.77 Mbp was adapter data, and the no-host data were 3,029.40 Mbp. The Q30 of the sequencing was 83.46%, the GC content was 42.94%, and the effective rate was 99.814%. However, only 1,177,922 reads (5.80%) were associated with viruses through comparisons of reads against the NCBI nonredundant nucleotide databases, reference viral protein databases and ACLAME database. These reads were further classified into three major orders, *Caudovirales* (684,931 reads, composition ratio 58.0%), *Picornavirales* (191,092 reads, 16.0%), and *Herpesvirales* (71,785 reads, 6.0%) (Fig 1A). At the family level, *Siphoviridae* (46.0%), *Myoviridae* (45.0%), and *Podoviridae* (8.0%) comprised most *Caudovirales*. *Picornaviridae* (99.9%) and *Herpesviridae* (99.0%) were the primary families of *Picornavirales* and *Herpesvirales*, respectively, as shown in Fig 1B. The unclassified order was divided into several families, such as *Microviridae* (47%), unclassified (36%), *Phycodnaviridae* (3%), *Mimiviridae* (3%), *Anelloviridae* (3%), *Inoviridae* (2%), *Polyomaviridae* (1%) and others (5%) (Fig 1B). Unknown/other reads indicated the proportion of highly divergent and/or novel sequences with no homology to NCBI.

**Fig 2:**
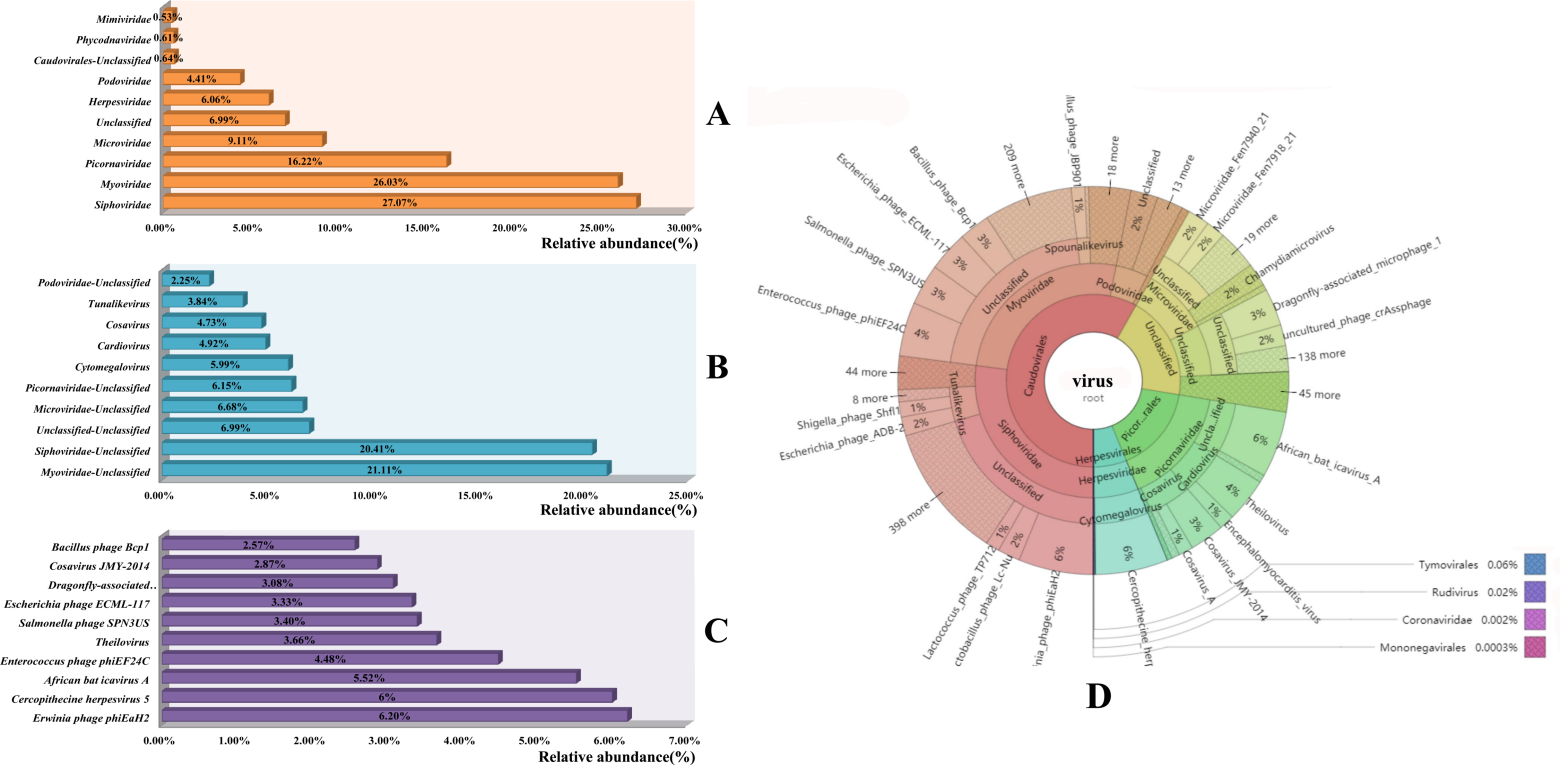
The relative abundance of the top 10 virus-related reads at different distribution levels and total taxonomic distributions. A. The bar graph of relative abundance for top 10 families in this study; B. The bar graph of relative abundance for top 10 genera in this study; C. The bar graph of relative abundance for top 10 species in this study; D. Total composition ratio of taxonomic distributions shown by Krona.

Based on the relative abundance of reads related to each classification, the top 10 families, genera and species of all reads were shown in Fig 2A–2C. The most relatively abundant family was *Siphoviridae* (composition ratio 27.07%), followed by *Myoviridae* (26.03%), *Picornaviridae* (16.22%), and *Microviridae* (9.11%) (Fig 2A). The top 5 genera were unclassified for each family, as shown in Fig 2B. *Cytomegalovirus* (composition ratio 5.99%), *Cardiovirus* (4.92%), *Cosavirus* (4.73%), and *Tunalikevirus* (3.84%) had a high relative abundance at the genus level. At the species level, high relative abundances were found for *Cercopithecine herpesvirus* (composition ratio 6.0%) and *African bat icavirus A* (5.52%); all others belonged to bacterial phages (Fig 2C). All the taxonomic distributions of the different levels of viruses were shown by Krona in Fig 2D.

According to the host types and nucleic acid classifications, all of the related viruses in this study were divided into bacterial phages (61.83%), animal-specific viruses (34.50%), plant-specific viruses (0.09%), insect-specific viruses (0.08%) and other viruses (3.50%) (Table 2). The double-stranded DNA viruses (dsDNA) accounted for 51.13% of the total, followed by single-strand RNA (ssRNA) (33.51%) and single-strand DNA (ssDNA) viruses (15.36%), as shown in Table 2. The data revealed a wide diversity of viruses with a prevalence of bacterial phages and described a range of animal viruses, such as ssRNA viruses belonging to the order *Picornavirales*, ssDNA viruses (*Circovirus*), and dsDNA viruses (*Adenovirus* and *Herpesvirus*). In addition, some of the results were related to insect viruses in *Densoviridae* and *Iridoviridae* and plant viruses in *Caulimoviridae* and *Potyviridae*.

**Table 2:** Distribution of different virus categories in the tree shrew gut virome.

	dsDNA^a^	ssDNA^b^	ssRNA+^c^	Subtotal	Perc.
	(Group I)	(Group II)	(Group IV)		
Animal virus	1663	1821	116782	120266	34.50%
Plant virus	276	0	45	321	0.09%
Phage	163810	51708	0	215518	61.83%
Insect viruses	295	0	0	295	0.08%
Other viruses	12184	0	0	12184	3.50%
Subtotal	178228	53529	116827	348584	
Perc.	51.13%	15.36%	33.51%		

^a^ The typical dsDNA viruses in this study were *Siphoviridae*, *Myoviridae*, *Poxviridae* (*Orthopoxvirus*), *Polyomaviridae* (*Alphapolyomavirus*), and *Adenoviridae* (*Mastadenovirus*) etc.

^b^ The typical ssDNA viruses in this study were *Microviridae* and *Inoviridae* etc.

^c^ The typical ssRNA virus in this study was *Picornaviridae* (*Cardiovirus*, *Cosavirus*, and *Mischivirus*) etc.

### Phylogenetic analysis

**Fig 3:**
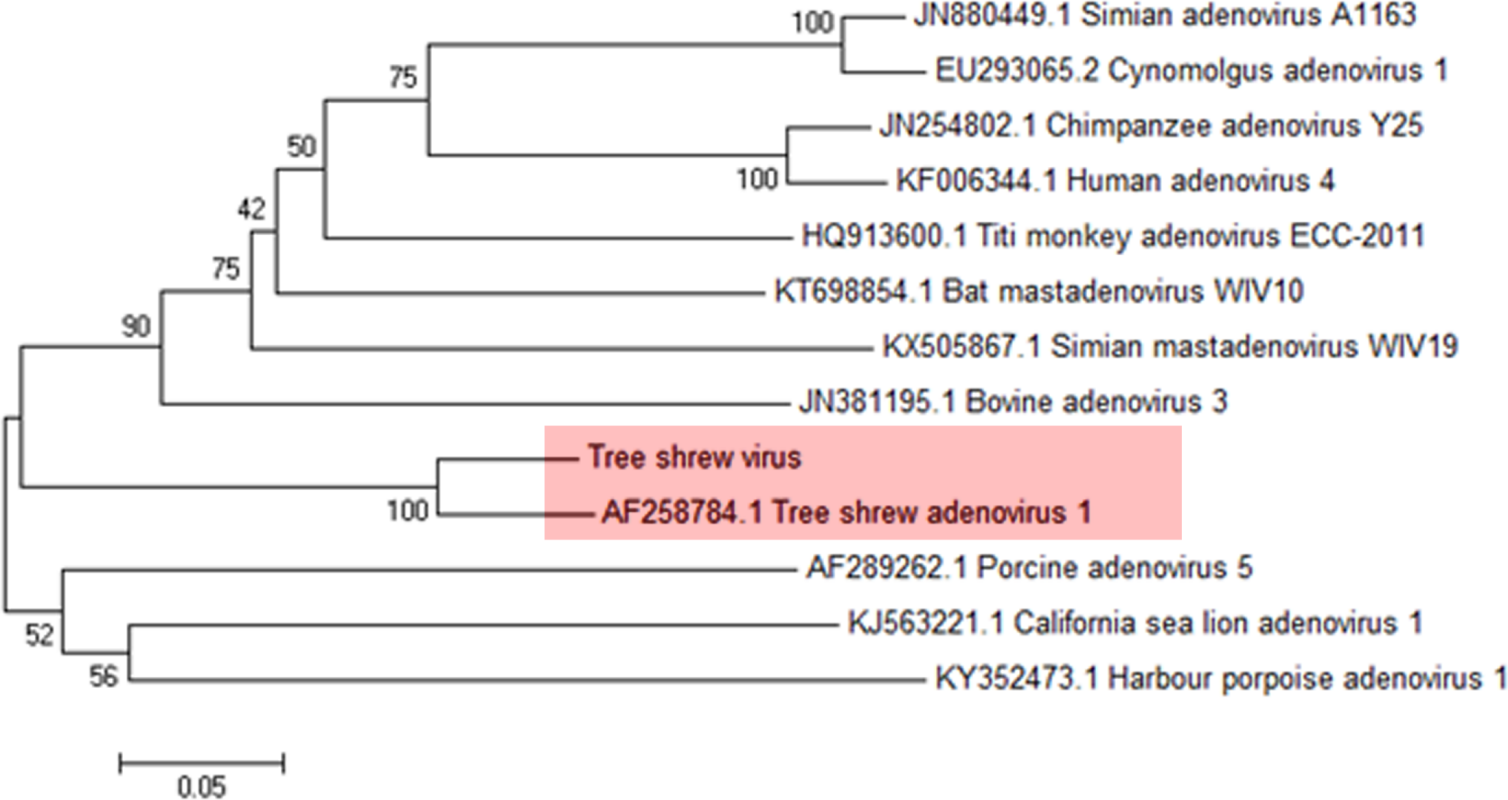
Phylogenetic analysis of the *3’UTR* region of adenovirus. The red area indicated the best match result between sequence in this study with reference adenovirus (AF258784.1).

To confirm the discovery of adenovirus, PCR assays were used to amplify the conserved region of the *3’UTR* gene of adenovirus. The results showed that the *3’UTR* of adenovirus in this study had 93% similarity to the newly described tree shrew adenovirus A (GenBank: AF258784.1) (Fig 3).

**Fig 4:**
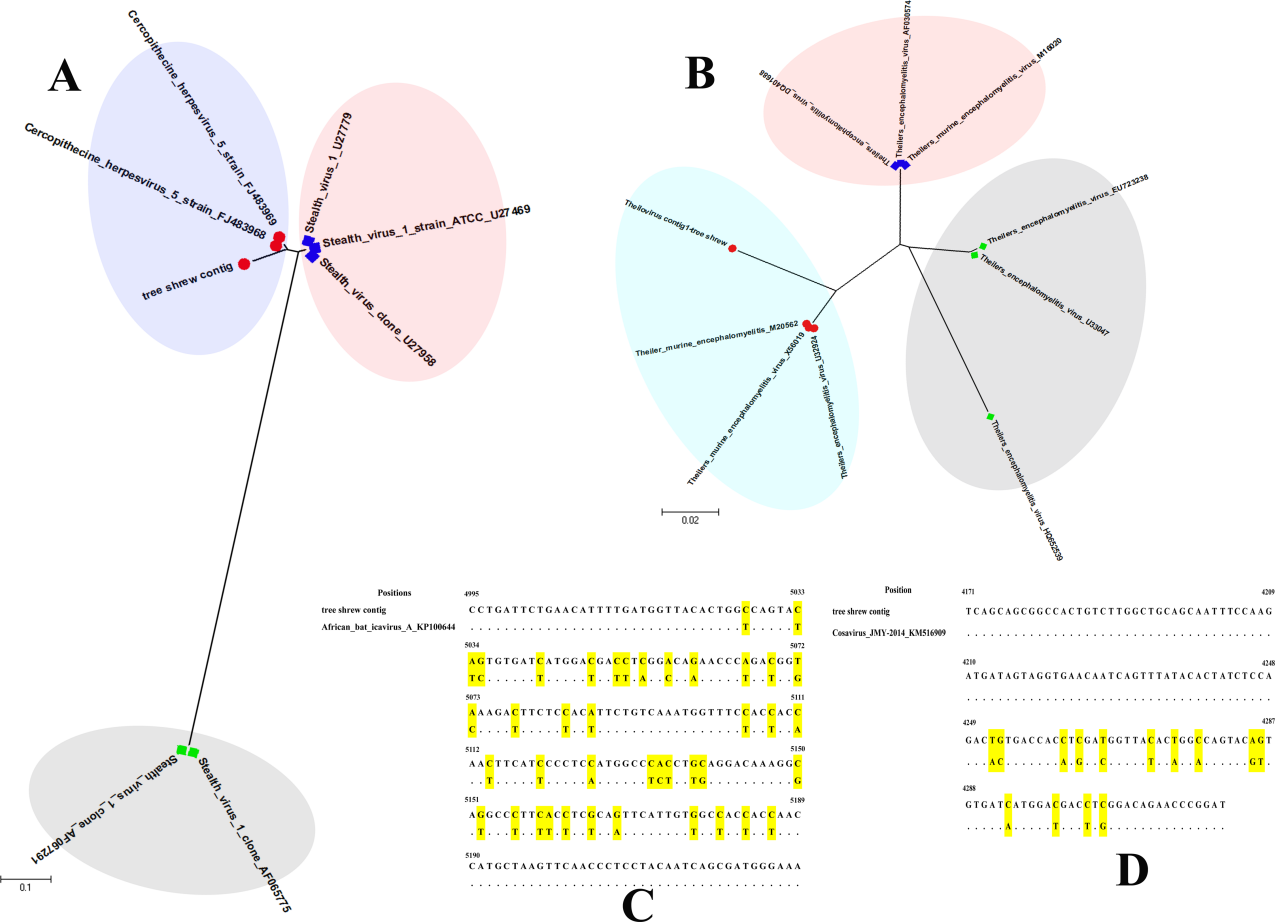
Phylogenetic analysis of the top virus species in the tree shrew gut virome. A.Phylogenetic tree of *Cercopithecine herpesvirus 5* with tree shrew contig; Three cluster groups were generated by using MEGA 6.0, and shown by different colors. B. Phylogenetic tree of *Theilovirus* with tree shrew contig; Three cluster groups were generated by using MEGA 6.0, and shown by different colors. C.The diagram of base substitutions between *African bat icavirus A* and tree shrew contig; Yellow areas indicated the positions of base changes. D. The diagram of base substitutions between *Cosavirus JMY-2014* and tree shrew contig.Yellow areas indicated the positions of base changes.

For the perspective phylogenetic analysis, the coverage of generated contigs of top virus species were above 80% for each reference genomes, and the details of the alignments were shown in S1 Fig. Part of the regions for each contig was selected to perform the phylogenetic analysis. The phylogenetic analysis of the tree shrew contig for *Cercopithecine herpesvirus 5* showed 93% similarity with reference NC_012783, ranging from 63,701 to 64,006 positions (306 bp) of the genome, and referred to the *UL48* (accession: FJ483968) gene. The second most similar virus with this contig was *Cercopithecine herpesvirus 5* strain Colburn (accession: FJ483969), which showed 92% similarity, ranging from 63,916 to 64,221 positions of the genome. These two gene with contigs composed a cluster in Fig 4A (blue area), and BLAST results (*Stealth virus*) formed two other groups, as shown in the pink and gray areas of Fig 4A. The tree shrew contig of *Theilovirus* showed 92% similarity with *Theiler’s murine encephalomyelitis virus* (TMEV) (accession: X56019), *Theiler's encephalomyelitis virus* (accession: U32924), and *Theiler’s murine encephalomyelitis* (accession: M20562). These viruses constituted a cluster (blue area) in Fig 4B. Other *Theiler’s encephalomyelitis virus* clustered in two groups in Fig 4B (pink and gray area), but the total similarity was above 84%. For tree shrew contigs of *African bat icavirus A* and *Cosavirus JMY-2014*, only one BLAST result was obtained. The contig of the tree shrew showed 83% similarity with *African bat icavirus A* (accession: KP100644), ranging from 4,995 to 5,223 positions of the genome, and several transitions or transversions were found by alignment analysis, as shown in Fig 4C (yellow area). A 91% similarity was identified between tree shrew contig and *Cosavirus JMY-2014* (accession: KM516909), ranging from 4,171 to 4,320 positions of the genome, and only 14 single base substitutions were discovered, as shown in Fig 4D (yellow area).

## Discussion

The viral metagenomics method has been employed to identify both commensal viruses and viral pathogens successfully in recent years and has the potential to detect most viruses through sequence similarity searches [11, 25]. Due to considerable genetic homology with both humans and primates, the tree shrew was considered to be a model for studies on viral infection and preclinical drug development [2]. This study indicated that the value of tree shrew as a model animal was increased because of the presence of a fecal virome.

A large number of studies have been performed on animal viromes, both in wild animals and in domestic animals [13, 26–27]. Ng et al. [28] conducted a wide survey of viral diversity within mosquitoes using metagenomics. Viral reads represented only 1% to 2% of total reads, and animal viruses represented not more than 10% of viral reads. As a consequence, animal viruses detected in mosquitoes may have reflected the virome of a large variety of vertebrate hosts (e.g., humans, primates, or birds). In our study, only 5.8% of reads exhibited significant similarity to the sequences deposited in the viral reference database. A total of 94% of sequences could not be classified, which was consistent with other metagenomic studies of fecal viromes. Several factors may lead to this phenomenon, such as limited representation of viruses in reference sequence databases, limitations of alignment-based classification, and the divergence or length of viral sequences [29]. We considered maybe it was due to the “Viral dark matter”, that only a small fraction of the total nucleic acids were known viral origins, and unknown sequences dominated viromes as 63%-93% of the reads often lack functional or taxonomic annotations [30]. Phan et al. [14] performed a metagenomic analysis of fecal specimens from mice, voles and rats. Their results showed that the presence of insect (e.g., *Densovirinae*, *Iridoviridae*) and plant viral sequences (e.g., *Nanoviridae*, *Geminiviridae*) reflected the diet of rodents. They also noted the presence of plant viruses, such as *Virgaviridae*, in the virome of the rodents’ feces. Similar results could be found in our study because insect- or plant-specific viruses were identified, which also reflected the eating habits or life cycles of tree shrew. Furthermore, the phylogenetic analysis of tree shrew contigs or genes showed high similarity with reference viruses, such as *Cercopithecine herpesvirus 5*, *Theilovirus*, *African bat icavirus A*, *Cosavirus JMY-2014* or *adenovirus*, possibly reflecting real infectants of the animal.

Hofer et al. [31] showed 87% of the contigs for human gut virome had no overlap with previously identified viruses, and 13% belonged to phage families, including *Microviridae*, *Podoviridae*, *Myoviridae* and *Siphoviridae*. Carding et al. [32] reviewed that the human intestinal virome was personalised and stable, and dominated by phages. The most distributed three families were *Siphoviridae*, *Myoviridae* and *Podoviridae*. For the non-human primates, D’arc et al. [33] evaluated the gorilla gut virome in association with natural simian immunodefciency virus infection. Their results showed that three bacteriophage families (*Siphoviridae*, *Myoviridae* and *Podoviridae*) represented 67.5 and 68% of the total annotated reads in SIVgor-infected and uninfected individuals, respectively. Specifically, the *Siphoviridae* family was more frequent in SIVgor-infected individuals compared with uninfected individuals, while two other bacteriophage families were more frequent in uninfected individuals. Liu et al. [34] performed the metagenomic analysis of wild rhesus monkey gut virome in China. Except for bacteriophage, five vertebrate virus families, six insect virus families and eleven plant virus families and other viruses were found in their study. All these studies showed similar results with ours, no matter for human or non-human primates. As alternatives to primates as lab animal models, the tree shrew gut virome was indeed comparable to current animal models.

Bacteriophages have biomedical importance because they can transmit genes to their bacterial hosts, conferring increased pathogenicity, antibiotic resistance, and new metabolic capacity. Previous studies have shown that siphophage fragments were the most common fragments observed in published metagenomic libraries. In particular, siphophages constituted 44% of phage sequences in the sediment library [25]. Viruses were present in several environments, which indicated that siphophages might be the most abundant genomes on Earth. In our study, the most abundant virus was *Siphoviridae*, reflected the distribution characters of tree shrew gut virome. In addition, we also found some specific viruses in the gut virome of tree shrew. Approximately 15.36% of the virome was detected as the ssDNA virus, the dominant virus; for example, *Mischivirus*, a pathogen that may cause human disease, was also found in the body of a bat [35]. For dsDNA viruses, the dominant genus, *Orthopoxvirus*, under the family *Poxviridae*, uses vertebrates, including mammals and humans, and arthropods as natural hosts [36–37]. Diseases associated with this genus include smallpox, cowpox, horsepox, and monkeypox. There were currently ten species in this genus, including the type species vaccinia virus, which was the dominant species (55%) of this category for animal viruses in the tree shrew gut virome. The second dominant genus, i.e., *Alphapolyomavirus*, under the family of *Polyomaviridae*, may infect humans and other mammals [38]. *Polyomaviridae* is a family of viruses whose natural hosts are primarily mammals and birds. Some members of the family, such as Merkel cell polyomavirus and raccoon polyomavirus, are oncoviruses known to cause tumors or cancers in their natural hosts [38]. The third dominant genus, i.e., *Mastadenovirus*, under the family of *Adenoviridae*, has human, mammal, and vertebrate natural hosts. Diseases associated with this genus included respiratory, gastrointestinal and eye infections, among others [39]. Furthermore, *Singapore grouper iridovirus*, under the family *Iridoviridae* [40] and categorized as an animal virus, has also been found in the tree shrew gut virome. All these results indicate that these viruses should be the focus of future studies involving disease model construction.

One major limitation of this study was pooling samples from 50 tree shrews to generate a single metagenome. The primary purpose of this research was to characterize the stool virome of tree shrew, and it would have been more informative to show variability of viruses detected and their proportional abundances across individuals, and to test whether virome composition correlated with host parameters (e.g. gender, body weight, health status). This would be the future investigation of tree shrew gut virome for our further study.

## Supporting information

S1 Fig The details of alignments for top virus species in this study. A. The diagram of reads mapping to reference *Cercopithecine herpesvirus 5*.
B. The diagram of reads mapping to reference *Theilovirus*.
C. The diagram of reads mapping to reference *African bat icavirus A*.
D. The diagram of reads mapping to reference *Cosavirus JMY-2014*.
(TIF)
