# Supplementary figures and images for: Characteristics of the tree shrew gut virome
Source: PLoS One. 2019 Feb 26;14(2):e0212774. doi: 10.1371/journal.pone.0212774 (PMC6391014; doi:10.1371/journal.pone.0212774)

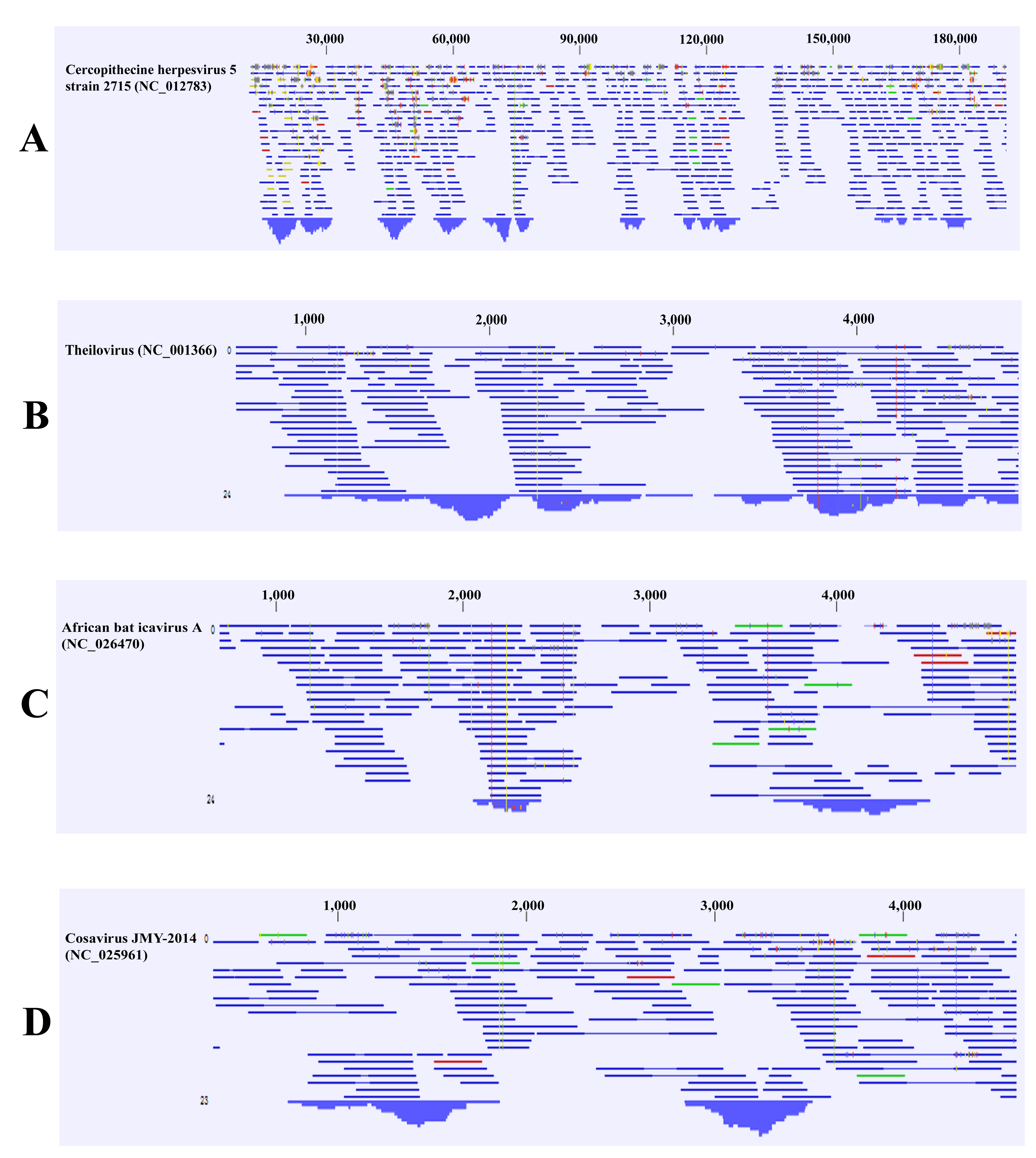

Supplement: S1 Fig — A. The diagram of reads mapping to reference Cercopithecine herpesvirus 5. B. The diagram of reads mapping to reference Theilovirus. C. The diagram of reads mapping to reference African bat icavirus A. D. The diagram of reads mapping to reference Cosavirus JMY-2014. (TIF) [file pone.0212774.s001.tif]
